# Screening Circulating Tumor Cells as a Noninvasive Cancer Test in 3388 Individuals from High-Risk Groups (ICELLATE2)

**DOI:** 10.1155/2018/4653109

**Published:** 2018-05-28

**Authors:** Juan Castro, Luis Sanchez, María Teresa Nuñez, Ming Lu, Tomas Castro, Hamid R. Sharifi, Christer Ericsson

**Affiliations:** ^1^Department of Oncology-Pathology, Karolinska University Hospital, Z1:00, 171 76 Stockholm, Sweden; ^2^iCellate Medical AB, Industrivägen 1, 171 48 Solna, Sweden; ^3^Hospital Nacional Arzobispo Loayza, Av. Alfonso Ugarte 848, Cl Javier Heraud 320, Urb Covima, La Molina, Lima, Peru; ^4^Department Pathology, Instituto Nacional de Enfermedades Neoplásicas (INEN), Av. Angamos Este 2520 Surquillo, El Condado St. 212 La Molina, Lima 12, Peru; ^5^Department of Microbiology, Tumor and Cell Biology (MTC), Karolinska Institutet, Biomedicum, Solnavägen 9, 171 65 Solna, Stockholm, Sweden

## Abstract

Cancer is known to spread up to 12 years before clinical symptoms occur, but few screening tests exist. Early detection would give the opportunity for early treatment, potentially improving prognosis. To this end, 3388 subjectively healthy individuals of age 45 to 80 who had been exposed to cancer risk factors were screened for the occurrence of circulating tumor cells in their blood. Presence of circulating tumor cells is a suspicious finding indicative of spreading cancer, since cancer metastasizes by way of the blood and offers the opportunities to (a) follow up the individual clinically based on established guidelines for early detection of cancer and (b) evaluate the cells further analytically. 107 individuals showed one or more circulating tumor cells in a 7.5 ml blood sample, which constitutes a positive circulating tumor cell test, based on the iCellate IsoPic™ laboratory test. That number compares favorably with the cancer incidence per 100,000 people/year that is 157.1 in Peru, given that a high-risk group of individuals was screened and that the screening results would be expected to correspond to an accumulated incidence of up to 12 years. The present findings therefore identify screening for circulating tumor cells as a promising new test.

## 1. Introduction

Epithelial cancers spread by inappropriately invading the basement membrane of extracellular matrix fibers, a key microscopic observation by clinical pathologists that forms part of the diagnosis of cancer. The invading cells may invade locally or may enter the lymph or blood vessels. The result is local or systemic spread of the cancer cells that may result in metastases. Clinical observation [1] and experimental testing [2] demonstrate that the spread can occur early in the disease progression, well before the primary tumor would give symptoms, be visible by medical imaging [3], or be detectable by serum tumor markers [3–5]. Screening for CTCs in the blood is therefore an inherently promising approach to early detection of cancer.

The first generation of CTC isolation instrumentation, which used the EpCAM cell surface biomarker as an immunoaffinity tag for cancer cell detection, followed up by verifying the epithelial nature of the detected cells by being DAPI-positive (i.e., having a cell nucleus), cytokeratin-positive (i.e., being epithelial), and CD45-negative (i.e., not a leucocyte), established the prognostic relevance of quantifying CTCs in 3 major late-stage cancers [6–8]. It was discovered that CTCs are extremely rare in healthy subjects and patients with nonmalignant diseases but present in various metastatic carcinomas with a wide range of frequencies [9]. Attempts to follow up on that initial promise for early detection of cancer were however disappointing [10, 11]. This may be related to the limitation of the cell surface marker used, EpCAM, since it may be lost during disease progression or depending on the state of the cells. In addition, genomic analyses of single CTCs remain a challenge to this day, although recent progress in sequencing technology offers new promise. The understanding now is that a more universal, marker-independent 2nd-generation approach to CTC isolation is required [10]. One such 2nd-generation CTC isolation instrument is the iCellate's IsoPic. In addition, it has previously been demonstrated that an IsoPic prototype could isolate CTCs from a patient with peritoneal carcinomatosis [12, 13]. The isolated cells could also be demonstrated to be genetically abnormal, consistent with being cancerous (Castro and Ericsson, unpublished data). The iCellate IsoPic would therefore seem to be a promising test to screen asymptomatic individuals for suspicious cells that can then be tested further, with the hope of the screening leading to the early detection of cancer.

In the present paper, we report the results of screening 3388 subjectively healthy individuals, with no cancer diagnosis, but with an increased risk of developing cancer based on age and having been exposed to other risk factors. We find 107 individuals carrying cells in their blood that satisfy the established criteria for CTCs. The iCellate IsoPic therefore seems to provide a promising cell sample for the early detection of cancer.

## 2. Results

**Figure 1:**
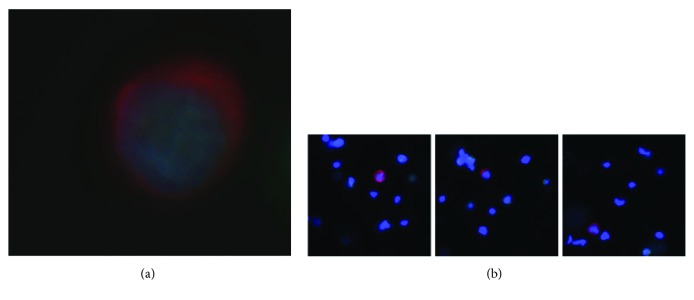
(a) A circulating tumor cell prepared from a 7.5 ml blood sample from a 79-year-old male with no previous history of cancer. The cell is stained for cytokeratin (red) and for the cell nucleus (blue), typical of epithelial cells. Epithelial cells should not normally be present in the blood. The cell was negative for CD45, that is, not an immune cell. The cell nucleus has a large size typical of transcriptionally active cells, such as cancer cells, and the rounded shape of a cell in suspension, rather than the angular shape and cell sheath context of a normal solid tissue epithelium cell. (b) Three additional examples of circulating tumor cells stained for cytokeratin (red) and for the cell nucleus (blue). The lower magnification also shows the residual leucocytes surrounding the circulating tumor cells (blue nuclei, with no cytokeratin (red). The samples were enriched about 7500-fold for CTCs, with about 10,000 DAPI and CD45-positive leucocytes left in the sample after enrichment.

3388 subjectively healthy people, with no previous history of cancer, were screened for the presence of any cells in a 7.5 ml blood sample that comply with the established definition of a CTC, such as the cells shown in Figure 1. Those cells were counted. 3281 subjects were negative for any such cells. 107 samples were positive for CTCs (see Table 1). The numbers of CTCs varied from 1 to 10. The positive patient samples were divided over the risk groups as follows: (1) heavy cigarette smoker (≥2 packets per day) more than 10 years: 12%; (2) family history of cancer: 82%; (3) chronic infection of hepatitis B virus (HBV) or hepatitis C virus (HCV): 1.5%; and (4) elevated prostate-specific antigen (PSA) level (≥4 ng/ml): 4.5%. The samples were enriched about 7500-fold for CTCs, with about 10,000 DAPI and CD45-positive leucocytes left in the sample after enrichment. The attending physician decoded any positive results, and a systematic recall program was initiated. This program is ongoing. The results suggest that screening for CTCs is already a valuable screening tool, whose merits will be further enhanced by further clinical follow-up and by molecular analyses of the isolated cells.

## 3. Discussion

The present study found that cells meeting the established criteria for CTCs can indeed be detected among subjectively healthy individuals with increased risk of cancer, suggesting those cells may be a promising new sample type for cancer health screening and early detection. These findings are in line with the clinical information that primary tumors spread metastases by way of the blood years before they give symptoms and can be diagnosed by medical imaging [1], but was by no means self-evident. The CellSearch technique, FDA-approved since 2004 for late-stage breast, prostate, or colorectal cancer, has not been able to meet expectations of establishing a screening test for spreading cancer. The discrepancy may be due to that the CellSearch method relies on a cell surface marker that may not be present on all cancer cells, making the IsoPic both more sensitive and more representative of the entire panorama of cancer cells [10].

The age-standardized cancer rate, incidence per 100,000 people/year is 157.1 in Peru, or about one-twentieth of the CTC incidence found in the present study. We think there are two reasonable explanations for the discrepancy between the clinical incidence of cancer and the incidence of CTCs: (1) this is a high-risk group in terms of age and four other risk factors, (2) since the spread of a tumor may occur up to 12 years [1] before it gives clinical symptoms, and given that the tumor spreads by means of CTCs, we think that up to 12 multiples of the yearly clinical incidence may be reasonable for the CTCs, based on the assumption that any cancer developing clinically one year later, two years later, and so on up to 12 years later would be discovered at any one given time by the CTC screen, but before symptom debut. A parallel study, with 1585 asymptomatic average-risk age-matched individuals, showed 27 samples positive for CTC, or 1.7% (manuscript submitted for publication). Thus, we think that the CTC findings entirely align with reasonable assumptions of the disease mechanism. Future clinical follow-ups, using established criteria for early cancer detection [14], and more detailed analyses of the CTCs will serve to further clarify the relationship between CTC incidence and clinical incidence.

The established criteria for identification of CTCs such as DAPI positivity, cytokeratin positivity, and CD45 negativity [15] demonstrate only that the isolated cells show traces of being epithelial and not of being an immune cell, but do not in and of themselves demonstrate that the cell is a cancer cell. Epithelial cells should however not be present in the circulation. Their very existence is therefore a warning sign and a suspicious finding that should trigger a clinical follow-up, since no CTCs should be present in circulation. Future clinical trials will establish the opportunities and limitations of this new CTC detection and quantification. In the meantime, since a screening test is not intended to be diagnostic on its own, individuals with suspicious findings must be referred to a physician for diagnosis and any consequent treatment [16]. Established criteria for the early detection of a variety of cancers exist [14]. Following up on a positive CTC test with established cancer early detection may be medically and health economically advantageous.

Previous experimental studies of cancer [17] have shown that only genetically abnormal cells can seed metastases, not similar cells with functionally normal genomes. To bring cell-based diagnosis of cancer to its logical conclusion therefore requires that the isolated cells are also subjected to either functional tests for the hallmarks of cancer [18, 19] or are genetically sequenced, followed by the relevant bioinformatics analysis, to establish their cancerous nature. Such tests, in conjunction with the isolation of the inappropriate cells, would be expected to enhance the clinical relevance of the CTC detection and quantification. It should therefore be clear that to go beyond a suspicious finding and towards establishing that the cells are actually cancerous, even if at a very early stage, will require genetic analyses of the cells. For those cases, the present sample type would provide an ideal sample.

It will also be of interest to compare the genetic analyses of isolated CTCs to those of free circulating tumor DNA, ctDNA, to establish the merits of each sample type. The ctDNA sample is easily available, but is degraded, diluted, and admixed with normal DNA and has lost its cellular context, but may still provide a useful alternative when those negative factors are of lesser importance. Since the CTCs are the actual vehicles of metastatic spread and provide a highly concentrated and pure sample in its cellular context, it would be expected that CTCs would remain the “gold standard” of blood-based cancer testing.

Summary: CTCs can be detected in asymptomatic individuals. A positive CTC-screening test is a suspicious finding that should be followed up clinically. The value of the test will be further enhanced following clinical trials and by adding new molecular tests of the isolated circulating tumor cells.

## 4. Methods

ICELLATE2 is an all-comer, single-center trial including individuals in the age of 45–80 years and belonging to any of the 4 risk groups: (1) heavy cigarette smoker (≥2 packets per day) more than 10 years, (2) family history of cancer, (3) chronic infection of hepatitis B virus (HBV) or hepatitis C virus (HCV), and (4) elevated prostate-specific antigen (PSA) level (≥4 ng/ml). Any individual with a current diagnosis or history of any types of tumor, HIV infection, or pregnancy was excluded.

All experimental protocols were approved by the Instituto Nacional de Salud, Comité de Investigación, Lima, Peru. The methods were carried out in accordance with the relevant guidelines and regulations. Informed consent was obtained from all subjects.

### 4.1. Blood Draw

The subjects provided 7.5 ml of blood for CTC analysis.

### 4.2. CTC Isolation

The cells are selected by a new microfluidic device, the IsoPic, in the process of being commercialized, which separates the cells based on their biomechanical properties: flow rate, surface interactions, plasticity, and elasticity. The details cannot be further disclosed for intellectual property right reasons. The CTC isolation was carried out within 24 h after the blood draw with iCellate's isolation technology according to the IsoPic operators' manual. The IsoPic system is intended to, from a blood sample, identify, concentrate, and purify CTC to make them available as a sample to support the enumeration and analyses to support diagnosis of metastatic tumors and those nonmetastatic tumors that may release circulating tumor cells.

### 4.3. Cell Staining

The cells were washed in PBS for 5 minutes and then permeabilized with 0.2% Triton X-100 in PBS for 5 minutes and then washed by addition of PBS for 5 minutes.

The antibody/DAPI mix was added and incubated for 1 hour in the dark.

The cells were then washed twice with PBS for 5 minutes and mounted in a hydrophilic mounting medium with antifade and covered with a coverslip without catching air bubbles. The cells were then viewed using a microscope. The following DAPI and antibody preparations were used: DAPI (4′,6-diamino-2-phenylindole dihydrochloride, Cat. number D9542-5mg, Sigma-Aldrich), anti-Pan cytokeratin (AE1/AE3), eFluor 570 (Cat. number 41-9003-82, eBioscience), and anti-human CD45 (2D1)-FITC (Cat. number 345808, BD).

### 4.4. Microscopic Evaluation

The microscope system was a Nikon Eclipse Ni-E equipped with a CoolLED pE-300 light source, a CFI Plan Fluor 20x objective, and a Hamamatsu ORCA-Flash4.0 camera. The cell selection criteria were 8–25 *μ*m cell diameter, cytoplasmic cytokeratin positivity, a DAPI-positive cell nucleus, and no CD45 reactivity. The NIS-Elements Advanced Research software package was used to assist the evaluation. The microscopic evaluation was carried out by two independent experienced operators.

## Figures and Tables

**Table 1:** Numbers of circulating tumor cells relative their age, for individual subjects. 1 to 10 circulating tumor cells per 7.5 ml blood sample in 107 individuals out of the 3388 screened.

Age	Total number pat	1 CTC	2 CTC	3 CTC	4 CTC	≥5 CTC	Number of CTC pos pat	%
45–49	699	6	12	3	3	2	26	3.72
50–54	734	3	12	4	4	2	25	3.41
55–59	674	1	7	6	1	1	16	2.37
60–64	518	2	10	3	1	1	17	3.28
65–69	374	0	8	1	0	1	10	2.67
70–74	230	0	4	3	0	1	8	3.48
75–80	159	0	4	0	0	1	5	3.14
	3388	12	57	20	9	9	107	3.16
